# Altered sucking dynamics in a breastfed infant with Down syndrome: a case report

**DOI:** 10.1186/s13006-020-00318-4

**Published:** 2020-08-15

**Authors:** Viviane Silva Coentro, Donna T. Geddes, Sharon L. Perrella

**Affiliations:** School of Molecular Sciences, The University of Western Australia, M310, 35 Stirling Highway, Crawley, 6008 Western Australia

**Keywords:** Breastfeeding, Sucking skills, Tongue movement, Down syndrome

## Abstract

**Background:**

The health and developmental advantages of human milk and breastfeeding are particularly important for infants with Down syndrome. However, they typically have shorter breastfeeding duration due to sucking issues that are not well understood. This case report describes serial measures of milk transfer volumes, sucking dynamics and tongue movement in a breastfeeding infant with Down syndrome. Management of maternal milk production enabled feeding of only breast milk until maturation of breastfeeding skills and the achievement of full breastfeeding by 6 months.

**Case presentation:**

The mother of a term infant with Down syndrome and no associated health complications presented with concerns regarding adequacy of milk removal at the breast and low milk supply. We monitored sucking dynamics during breastfeeding by measuring intraoral vacuum strength, nutritive and non-nutritive suck rates and burst durations, and tongue movement using submental ultrasound. Breastfeeds were monitored at 4, 10, 14, 19 and 24 weeks, and maternal 24 h milk production was measured at 4, 10 and 24 weeks postpartum. We observed a weaker suck strength and shorter nutritive suck duration, and atypical tongue movement up to 19 weeks, with low milk transfer volumes. Regular breast expression was effective in increasing maternal milk production, providing expressed milk for all complementary feeds. Full breastfeeding was achieved by 6 months when reference sucking values were observed.

**Conclusions:**

This case report illustrates that infants with Down syndrome may have low intraoral vacuum and limited nutritive sucking that persists for several months, likely due to delayed oro-motor development. In the absence of effective sucking human milk feeding can continue when milk production is stimulated with frequent and adequate breast expression. It is possible for infants with Down syndrome and no associated health complications to eventually establish full breastfeeding. Mothers that wish to breastfeed their infant with Down syndrome require anticipatory guidance and continuing lactation and family support.

## Background

Down syndrome (Trisomy 21) is the most common chromosomal disorder with a reported incidence of 1 in 1100 Australian births, or 270 births per year [1]. Although the life expectancy of people with Down syndrome has increased over the last 30 years [2], the syndrome is associated with global developmental delay and impaired intellectual, immune, thyroid and sensory functions while congenital heart disease, gastrointestinal disorders, otitis media and obesity are common [3]. A relatively large tongue, hypotonia and atypical oro-motor development can contribute to speech and feeding difficulties with disorganised and dysfunctional sucking patterns reported [4–6]. For infants and children with Down syndrome and clinical signs of eating or drinking difficulty or suspected aspiration, the incidence of pharyngeal dysphagia and oral dysphagia is as high as 58 and 64% respectively [5, 7]. Silent aspiration and the risk of pneumonia is significantly higher in this population [5, 8]. Breastfeeding is protective against respiratory and ear infections [9] and provides cognitive benefits [10]. As human milk has substantial beneficial impacts on health and development both in infancy and later in life, it is essential for optimisation of the lifetime health of infants with Down syndrome.

Shorter breastfeeding duration is reported for infants with Down syndrome, with a mean duration of 7.7 weeks compared to 23.4 weeks in healthy infants. Maternal reasons for weaning infants with Down syndrome include perceived low milk supply and poor infant suck [11]. Feeding difficulties are common and may be related to anomalies of oro-facial and naso-maxillary anatomy with altered reflexes and perioral hypotonia potentially impacting attachment and milk transfer at the breast [12].

Adequate intra-oral vacuum is fundamental to successful breastfeeding [13] yet there is no published data for sucking dynamics in breastfeeding infants with Down syndrome. Bottle fed infants with Down syndrome have a weaker suck and shorter suck bursts than age matched controls [14]. However sucking pressures, frequencies and efficiency are reported to improve over the first 12 months. Sucking dynamics differ between bottle and breastfeeding so these findings cannot be directly applied to the breastfed infant [15]. An understanding of the progression of feeding skills in infants with Down syndrome is critical to the development of evidence-based guidelines to support breastfeeding dyads. We present a longitudinal case report of breastfeeding characteristics and sucking dynamics in an infant with Down syndrome in the first 6 months.

## Case presentation

The male infant was born at 38 weeks of gestation to a 32 year old primiparous mother. He developed respiratory distress within an hour of birth and was transferred to the neonatal intensive care nursery for treatment with supplemental oxygen. The infant was fed colostrum until resolution of respiratory symptoms on day two, when breastfeeding was initiated with use of a nipple shield to aid attachment. Three hourly breastfeeds were supplemented with infant formula or expressed breast milk (EBM) and he was exclusively fed breast milk from day four. The parents were informed of the infant’s clinical features of Down syndrome on the night of his birth and diagnosis was confirmed by genetic testing on day six. Associated health complications including congenital cardiac anomalies, thyroid disease and gastrointestinal malformations such as Hirschsprung’s disease were not identified [3].

The family was discharged home with instructions to breastfeed and supplement with EBM every 3 h. At 3 weeks the mother was not confident of adequate milk transfer at the breast and concerned about continued use of a nipple shield to maintain attachment at the breast. She felt that her milk supply was reducing and sought help from an international board certified lactation consultant. After measurement of 24 h milk production, informed written consent was provided to participate in a longitudinal study of infant sucking dynamics, milk production and transfer. Studies were conducted at 4, 10, 14, 19 and 24 weeks.

At 4 weeks the infant was breastfeeding with a nipple shield and supplemented with EBM after most feeds. The infant was sleepy during both breast and bottle feeding. Chin support and dancer hand techniques [16] had been trialled to aid feeding but were not found to be helpful. Simultaneous breast expression was performed twice daily using a hospital grade electric breast pump. The 24 h milk profile confirmed low supply ie. < 600 mL/24 h [17] so expression frequency was increased. Nipple shield use was discontinued at 8 weeks due to a maternal sensation of nipple tightness.

**Fig. 1:**
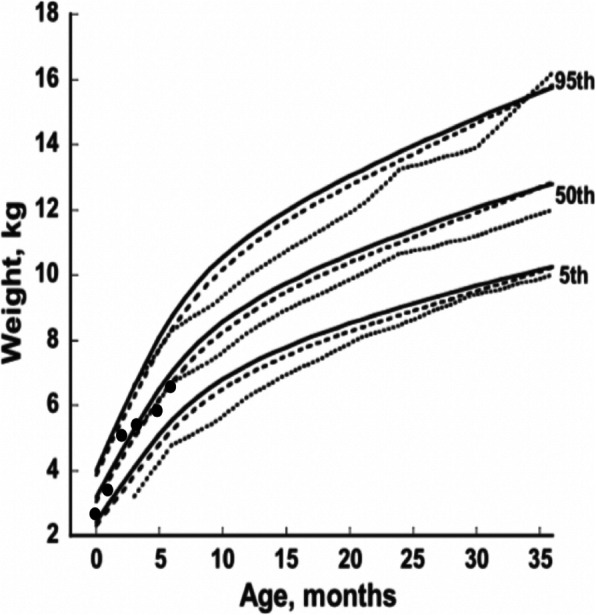
Weight-for-age chart for a male infant with Down syndrome (Birth to 6 months). Legend: The infant’s weight-for-age is plotted using black circles on growth curves for male children with Down syndrome from birth to 36 months of age. Contemporary growth curve is indicated by the solid line; previous published growth curves are indicated by a dotted line [18] and dashed line [19]. Growth curve chart is reproduced with permission from Dr. Babette Zemel [20]

As the mother was not confident of adequate milk transfer at the breast, an electronic baby scale was loaned so that the family could use test weights to guide supplementary feed volumes. Expressed breast milk supplements were given until 5 months. The mother aimed to feed the infant 800 mL/24 h. Using this strategy the infant maintained a pattern of weight gain considered adequate for male infants with Down syndrome (Fig. 1) [20]. The mother reported that test weighing was useful.

At the first visit, weak lip closure and compression without the application of intraoral vacuum was noted during digital examination. Sucking, rooting, gag and tongue thrust reflexes were present. The infant became fatigued during breastfeeding, but maintained an adequate seal and no swallowing difficulties were observed at all monitored breastfeeds. The mother reported that other than rare episodes of gagging, she did not observe any difficulties with swallowing. A speech therapist assessed the infant at 6 months and advised there were no signs of oral phase or pharyngeal phase dysphagia so family foods could be introduced.

The infant was regularly reviewed by a paediatrician and was treated for concurrent episodes of otitis media and urinary tract infection at 3 months. He was otherwise well, maintained adequate weight gain and had no clinical signs associated with aspiration and so was not investigated for this.

At 24 weeks the dyad was exclusively breastfeeding. The mother was confident of her infant’s milk intake and enjoyed her breastfeeding experience. She now felt certain of achieving her initial goal of breastfeeding for 12 months. Twice daily breast expression continued for the purpose of storing EBM for later use. First foods were gradually introduced from 6 months and breastfeeding continued until mutual weaning at 19 months.

## Summary of study findings

The 24 h milk profile was obtained by recording infant test weights before and after each feed from each breast on an electronic scale (BabyWeigh, Medela Inc., McHerry IL, USA, resolution 2 g, accuracy ±0.034%), and volumes of milk expressed and fed over a 24 h period [17]. Milk transfer volumes were recorded at each study visit [21]. Measurements expressed in grams were considered equivalent to mL [22]. While breastfeeding frequency and duration remained within the reference range, mean transfer volumes were low thereby requiring supplementation until 24 weeks (Table 1).

**Table 1:** Feeding characteristics in a breastfeeding infant with Down syndrome at 4, 10 and 24 weeks

	4 weeks	10 weeks	24 weeks	Reference range [17, 23]
24 h milk production (mL)	546	819	1150	788 ± 169
24 h breastfeed frequency	12	12	14	11 ± 1.6
Breastfeed duration (min)	11	16	14	16 (13, 23.6)
Breastfeed intake/feed (mL)				
Left breast	25 (14, 54)	40 (9, 92)	72 (2, 118)	84 ± 28^a^
Right breast	19 (12, 28)	32 (10, 56)	60 (16, 114)	67 ± 26^b^
24 h expression frequency	6	7	0	
24 h expression volume (mL)	278	392	226	
24 h infant milk intake (mL)				
Breastfeeding	268	427	924	
Expressed milk	219	270	0	
Total milk intake	487	697	924	
Infant weight (g) (Birthweight = 2700 g)	3478	5006	6425	

Feeding characteristics reported as mean ± standard deviation or median (min, max) for 24 h reference range, breastfeed duration and milk intake for left and right breasts. Measured values reported for the 24 h milk production, 24 h breastfeed frequency, 24 h expression frequency, 24 h expression volume and infant weight at monitored breastfeeds

^a^Values for more productive breast, ^b^Values for less productive breast

The 24 h milk production was low at 4 weeks and increased with regular breast expression. The infant received only breast milk through breastfeeding and EBM supplementary feeds from day 4 to 6 months of age, when consistent adequate milk transfer was achieved, allowing full breastfeeding.

Intra-oral vacuum was measured as previously described [13] with graphical representation of intra-oral vacuum used to measure sucking rate, bursts and pauses during monitored breastfeeds. Weak baseline and peak intra-oral vacuums were observed up to 19 weeks and were weaker with nipple shield use at 4 weeks. Reference value intra-oral vacuums, typically achieved by 4 weeks of age, were observed at 24 weeks (Table 2). Nutritive suck burst durations were shorter than reference up to 19 weeks, with a faster nutritive suck rate persisting across the first 6 months (Table 2) [23].

**Table 2:** Sucking dynamics at 4, 10, 19, and 24 weeks in an infant with Down syndrome

Infant age	4 weeks	4 weeks	10 weeks	14 weeks	19 weeks	24 weeks	Reference range
**Breast**	**left**	**right (shield)**	**right**	**left**	**right**	**left**	**average**
Peak vacuum (mmHg)	− 59 ± 33.5	− 43 ± 29	−38.5 ± 20	−54 ± 35	−58 ± 40	− 133 ± 43	− 145 ± 58 [13]
Baseline vacuum (mmHg)	−6 ± 8	−2 ± 5	0 ± 4	−6.5 ± 11.5	−12 ± 18	−31 ± 30	− 64 ± 45 [13]
NS burst duration (s)	3.3 (0.4, 8)	2.3 (1, 10)	3.8 (0.6, 19)	3.7 (0.5, 17.5)	4.5 (0.7, 20)	6.0 (0.4, 75)	8.9 (4.5, 18.3) [23]
NNS burst duration (s)	2.0 (0.5, 2.7)	2.7 (2, 8)	1.6 (0.6, 7.5)	2.5 (0.5, 10.5)	5.0, (1, 10.5)	2.8 (0.6, 29.5)	4.5 (3.1, 7.1) [23]
NP duration (s)	2.3 (0.6, 35)	3.0 (0.5, 41.2)	1.4 (0.3, 19)	1.0 (0.6, 76.5)	0.9 (0.5, 9.1)	0.7 (0.4, 99.6)	2.9 (1.8, 5.5) [13]
NNP duration (s)	3.5 (1.7, 13)	16 (2.6, 48.7)	2 (0.3, 37.5)	2 (0.7, 128.2)	18 (0.9, 67)	2.3 (0.6, 19.5)	2.7 (1.9, 4.0) [13]
Milk intake (mL)	22	4	42	42	48	125	76 (30–135) [17]
NS rate (sucks/min)	101 ± 22	110 ± 31	87 ± 17	96 ± 27.5	97 ± 22.5	105 ± 22.7	74 ± 17.1 [23]
NNS rate (sucks/min)	85.4 ± 19	112.3 ± 22	93 ± 28.5	105.4 ± 20.3	91.4 ± 13	118 ± 33.2	88.9 ± 23.9 [23]
Feeding efficiency (mL/min)	4.7	1.5	6	6	8	11.5	8.9 ± 4.2 [24]

Sucking dynamics reported as mean ± standard deviation or median (min, max) for peak and baseline vacuums, milk intake, nutritive (NS) and non-nutritive suck (NNS) rate and burst duration, nutritive (NP) and non-nutritive pause (NNP) duration and feeding efficiency at monitored breastfeeds for an infant with Down syndrome at 1–6 months of age

**Fig. 2:**
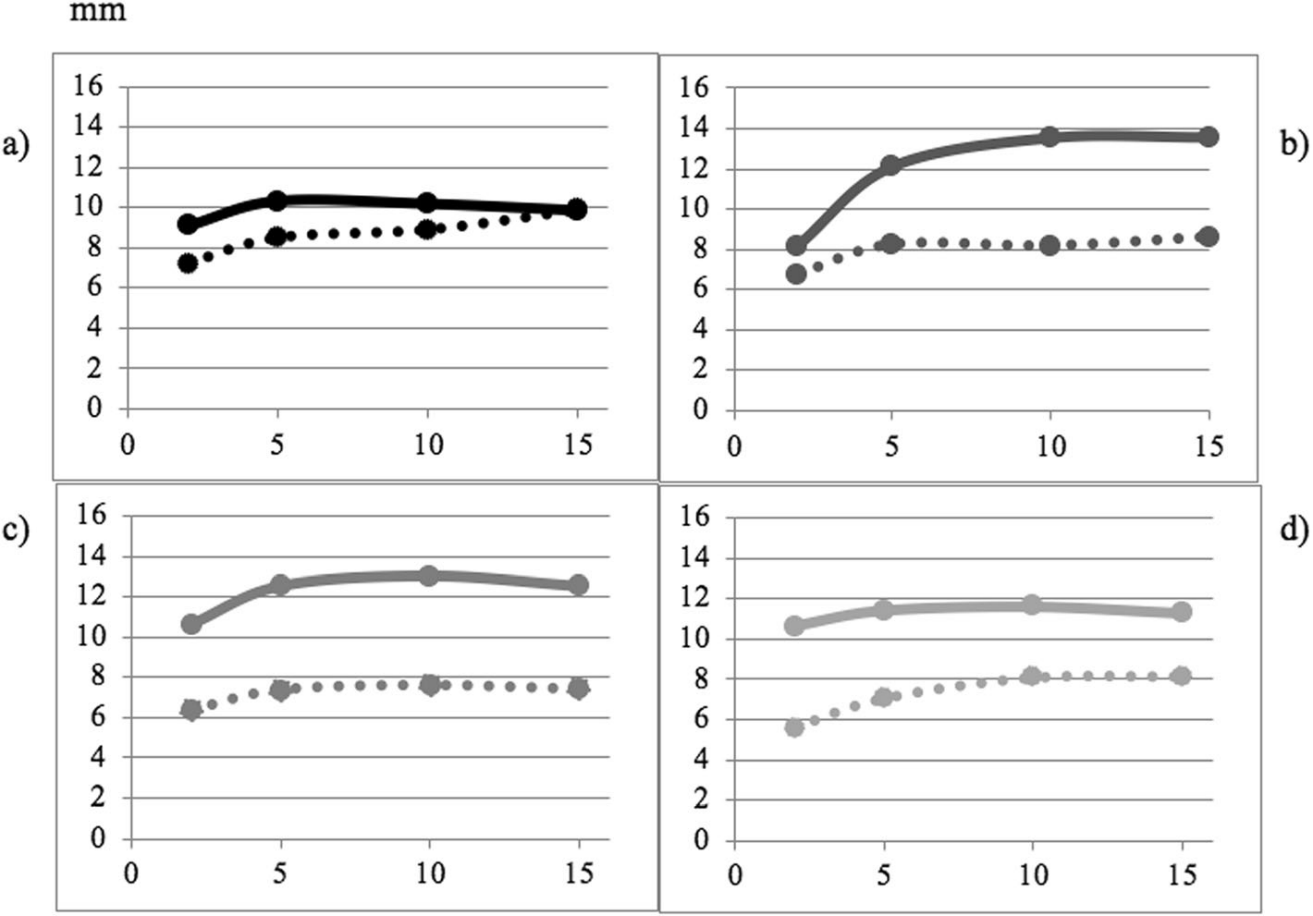
Tongue movement at 4, 10, 19 and 24 weeks in an infant with Down Syndrome. Legend: Measurements taken at **a** 4 weeks **b** 10 weeks **c** 19 weeks and **d** 24 weeks. Tongue up: solid line; tongue down: dotted line

Sub-mental ultrasound imaging of the infant’s intra-oral cavity was performed at each study visit enabling a clear view of the nipple, tongue, hard palate, soft palate, and milk flow [13, 25]. All ultrasound scans were performed for the duration of the breastfeed, beginning when the infant first attached to the breast. Ultrasound image measurements were made at tongue-up and tongue-down phases of nutritive sucking as described by McClellan [25]. Nipple placement was determined by measurement of the nipple hard-soft palate junction (HPSPJ). The HPSPJ distance was not different and within reference range for tongue-up (5.6, 5.7, 6.2, 6.9 mm) and tongue-down (3.9, 3.9, 3.8, 4.5 mm) at 4, 10, 19 and 24 weeks respectively indicating that attachment was maintained throughout suck bursts [25]. Reduced tongue movement was seen at 4 weeks, but not at subsequent visits (Fig. 2) where low intraoral vacuum was measured despite adequate downward displacement of the tongue.

## Discussion and conclusions

This case report described low milk transfer volumes associated with low intra-oral vacuum and shorter nutritive sucking bursts up to 20 weeks of age in a breastfed infant with Down syndrome. Exclusive breastfeeding was achieved by 24 weeks indicating a relationship between intra-oral vacuum, nutritive suck burst duration and milk transfer [23]. While the biomechanics of breastfeeding differ from that of bottle-feeding, our findings concur with the reported measures of low intra-oral vacuum and feeding efficiency that increased over the first 8 months in fourteen bottle fed infants with Down syndrome [14]. Adequate breast attachment was maintained despite low baseline and peak vacuums so it is unlikely that external efforts to achieve or maintain attachment would be helpful. It has been established that infant intra-oral peak vacuum or negative pressure, is the primary mechanism of effective milk removal during breastfeeding. Generation of intra-oral vacuum increases with inferior displacement of the tongue and mandible [26]. We observed limited displacement of the tongue at 4 weeks but not subsequent to this. Weak intra-oral vacuum in the infants with Down syndrome has been attributed to oral hypotonia [12] which may impede the infant’s ability to form an adequate seal with negative pressure (baseline vacuum) to facilitate milk transfer [14]. In a mouse model, when compared to non-syndromic pups those with Down syndrome had reduced contractility in two oro-motor muscle groups that are involved in mandibular movement, with some improvement in only one group of muscles over time [27]. For newborn infants with Down syndrome weaker intra-oral vacuum may be explained in part by reduced contractility of the oris orbicularis, masseter and/or buccinator muscles. It is likely that muscle strength and therefore mandibular oscillation and suck burst duration increase with enhanced neural recruitment and improved neuromuscular function that accompanies repeated use through feeding [28].

In the absence of an accurate clinical assessment tool for intra-oral vacuum during breastfeeding the perceived strength of suck on a gloved finger is used clinically. However, this may not reflect the infant’s nutritive sucking action and strength during breastfeeding. The 24 h milk profile provides an accurate measure of milk transfer and production, and test weights at home can be used to guide supplementation. Indeed, regular test weighs of preterm infants during the establishment of breastfeeding is associated with earlier attainment of exclusive breastfeeding [29] and so may be a useful strategy for breastfeeding mothers of infants with Down syndrome.

Effective milk removal from the breast results from a combination of the application of adequate negative pressure and the positive pressure of milk ejection [13, 30, 31]. Both a higher degree of fullness of the breast and stronger negative pressure are associated with higher milk transfer volumes at the breast [32], so for infants with low intra-oral vacuum higher milk volume transfers can be facilitated with a higher 24 h milk production. Regular breast expression will be required to ensure adequate milk production and therefore promote breast fullness until maturation of infant sucking dynamics.

Infants with Down syndrome may have reduced breastfeeding effectiveness with delayed attainment of adequate intra-oral vacuum and suck burst duration. As regular breast expression and supplementation may be required for an extended period until typical sucking dynamics are achieved, anticipatory guidance and continuing support will likely benefit mothers that wish to breastfeed their infant with Down syndrome. Further studies of breastfeeding dynamics in infants with Down syndrome will elicit the range of developmental progression of intra-oral vacuum and suck burst duration in this population.

## Data Availability

All data generated or analysed during this study are included in this published article (and its supplementary information files).

## Abbreviations

EBM: Expressed breast milk
h: hour(s)
HPSPJ: Hard palate soft palate junction
min: minute(s)
mL: millilitre
mm: millimetre
mmHg: millimetre of Mercury
NNP: Non-nutritive pausing
NNS: Non-nutritive sucking
NP: Nutritive pausing
NS: Nutritive sucking
s: second(s)
TU: Tongue up
TD: Tongue down
WHO: World Health Organization
